# Prognostic significance of early acute kidney injury in COVID-19 patients requiring mechanical ventilation: a single-center retrospective analysis

**DOI:** 10.1080/0886022X.2023.2205954

**Published:** 2023-05-03

**Authors:** Michal Sitina, Vladimir Sramek, Martin Helan, Pavel Suk

**Affiliations:** a Department of Anesthesiology and Intensive Care Medicine, St. Anne’s University Hospital Brno, Brno, Czech Republic; b Biostatistics, International Clinical Research Center, St. Anne’s University Hospital Brno, Brno, Czech Republic; c Faculty of Medicine, Masaryk University, Brno, Czech Republic; d Intensive Care Research, International Clinical Research Center, St. Anne’s University Hospital Brno, Brno, Czech Republic

**Keywords:** COVID-19, acute kidney injury, renal replacement therapy, early kidney dysfunction

## Abstract

Acute kidney injury (AKI) is associated with impaired outcomes in critically ill COVID-19 patients. However, the prognostic significance of early AKI is poorly described. We aimed to determine whether AKI on admission to the intensive care unit (ICU) and its development within the first 48 h predict the need for renal replacement therapy (RRT) and increased mortality. An analysis of 372 patients with COVID-19 pneumonia requiring mechanical ventilation without advanced chronic kidney disease from 2020 to 2021 was performed. The AKI stages on ICU admission and Day 2 were determined using adapted KDIGO criteria. The early development of renal function was assessed by the change in AKI score and the Day-2/Day-0 creatinine ratio. Data were compared between three consecutive COVID-19 waves and with data before the pandemic. Both ICU and 90-day mortality (79% and 93% vs. 35% and 44%) and the need for RRT increased markedly with advanced AKI stage on ICU admission. Similarly, an early increase in AKI stage and creatinine implied highly increased mortality. RRT was associated with very high ICU and 90-day mortality (72% and 85%), even surpassing that of patients on ECMO. No difference was found between consecutive COVID-19 waves, except for a lower mortality in the patients on RRT in the last omicron wave. Mortality and need for RRT were comparable in the COVID-19 and pre-COVID-19 patients, except that RRT did not increase ICU mortality in the pre-COVID-19 era. In conclusion, we confirmed the prognostic significance of both AKI on ICU admission and its early development in patients with severe COVID-19 pneumonia.

## Introduction

A high incidence of renal impairment with variable outcomes has been documented in COVID-19 patients [1–7]. SARS-CoV-2 seems to cause more acute kidney injury (AKI) than other respiratory infections requiring hospitalization [8]. Although primary impairment of renal function through the direct invasion of SARS-CoV-2 into kidney cells is probable [9], secondary factors such as hemodynamic alteration or sepsis contribute to its occurrence as well [10–13].

This study retrospectively analyzed the outcome data of mechanically ventilated COVID-19 patients hospitalized between March 2020 and December 2021 in the ICUs of the Department of Anesthesiology and Intensive Care Medicine, St. Anne’s University Hospital in Brno, Czech Republic. The department serves as a tertiary referral center and a primary regional ECMO center and takes care of the most severe COVID-19 patients.

We specifically aimed to determine if AKI and its early progression during the first 48 h after ICU admission are predictive of outcome and use of renal replacement therapy (RRT). Although numerous studies have shown the association between AKI on ICU admission and mortality in critically ill COVID-19 patients [14–19], none of them focused explicitly on the prognostic significance of the very early development of renal impairment after ICU admission.

## Methods

A retrospective analysis of the electronic medical records of all 458 COVID-19 patients hospitalized in our ICU between March 2020 and December 2021 was performed. COVID-19 patients not requiring mechanical ventilation (MV) (*n* = 17), patients requiring MV for less than 24 h or who died within 24 h after admission (*n* = 6), patients without COVID-19 pneumonia as the main cause of admission (*n* = 53) and patients with a history of advanced chronic kidney disease (CKD 3–5; *n* = 10) were excluded from the analysis. Thus, 372 patients with COVID-19 pneumonia requiring mechanical ventilation for more than 24 h without advanced CKD were further analyzed. Eighty-six patients (23%) required treatment with extracorporeal membrane oxygenation (ECMO). We applied no age criteria for inclusion. SARS-CoV-2 infection was confirmed with polymerase chain reaction (PCR) in all patients.

In most patients, the baseline creatinine prior to hospital admission, which was necessary to determine the stage of AKI, was not available. Therefore, for all patients, we calculated the expected normal creatinine level using the MDRD equation, taking gender and age into account and assuming 100 mL/min as the normal glomerular filtration rate [20–22]. We used the ratio of the measured creatinine level to the expected normal value to determine the AKI stage, which was classified according to the adapted KDIGO criteria [20,23], with ratios of 1.5–2.0, 2.0–3.0 and above 3.0 or the need for RRT defining AKI stages 1, 2 and 3. The other KDIGO criteria for AKI staging, namely, the absolute increase in creatinine or urine output, were not applied, as explained in the Discussion. In addition, we calculated the Day 2-to-Day 0 (D2/D0) creatinine ratio to assess kidney function development in the first 48 h of the ICU stay. We defined improvement, stationarity, and deterioration of kidney function as D2/D0 values under 0.8, 0.8–1.2 and above 1.2, respectively. To assess the burden of comorbidities, the Charlson comorbidity index (CCI) was calculated [24].

The AKI data of the patients with SARS-CoV-2 in three consecutive COVID-19 waves were compared. In addition, the AKI data of the COVID-19 patients were compared with the data of a cohort of 303 nonselected ICU patients without a history of advanced CKD who were hospitalized in our ICU in 2019, i.e., before the COVID-era, and required mechanical ventilation for more than 24 h.

Statistical analysis was performed using R software Version 4.0.0 [25]. Count data are presented as absolute numbers and percentages, and quantitative variables are presented as medians and interquartile ranges (IQRs). Differences in proportions were assessed by Fisher’s exact test. Differences in proportions among more than two levels of a categorical variable, e.g., mortality difference among three COVID-19 waves, were assessed with the Fisher’s exact test for 2-times-k contingency tables [26]. Differences in quantitative variables were assessed with the Mann–Whitney test or Kruskal–Wallis test. Kaplan–Meier survival analysis and log-rank tests were performed to assess the impact of AKI stage on admission on ICU mortality, 90-day mortality, and the use of RRT. There were no patients with censored data within 100 days of ICU admission. A Cox regression model was used to adjust the impact of AKI on admission on the ICU mortality, 90-day mortality and the use of RRT for age, sex, BMI and CCI, which may have an impact on the association. AKI stage on ICU admission was treated as a continuous variable. Similar Kaplan–Meier, and Cox regression analyses were performed to assess the impact of change in AKI stage and change in creatinine from Day 0 to Day 2.

The study protocol was approved by the ethics committee of the St. Anne’s University Hospital, Brno, and conducted in accordance with the ethical guidelines of the Declaration of Helsinki. The data were deidentified prior to analysis. The ethics committee waived the requirement for informed consent because the study only applied a retrospective analysis of anonymized data.

## Results

### Patient characteristics

A cohort of 372 patients was analyzed. As shown in Table 1, the age of the patients was 61.0 (17.3) years old, they were predominantly males (76.6%) and overweight with BMI 29.4 (8.7). On admission, the oxygenation index of the patients without the need for ECMO was 92.7 (54.5) mmHg. Seventeen percent of the patients were smokers. The most prevalent comorbidities were arterial hypertension and diabetes mellitus. The CCI was 2.0 (2.0), and it was higher for patients with AKI (*p* = 0.004). Ninety-two percent of the patients required vasopressors since ICU admission. The ICU and 90-day mortality were 38.4% and 50.0%, respectively. Sixty patients were admitted from the emergency department, 134 from hospital wards or HDUs and 178 were transferred from other hospitals. ICU (45%, 36% and 38%) as well as 90-day mortality (50%, 55% and 46%) did not depend on the source of ICU admission. ICU nonsurvivors tended to stay in the ICU longer, with lengths of stay of 15 (11) and 13 (10) days for the nonsurvivors and survivors, respectively, *p* = 0.06.

**Table 1.** Baseline characteristics of the patients.

	All	no AKI	AKI Stage 1	AKI Stage 2	AKI Stage3	
Number	372	297	33	28	14	
Age	61.0 (17.3)	60.0 (17.3)	67.0 (17.3)	65.0 (17.3)	68.0 (17.3)	years
Male gender	76.6	75.1	75.8	92.9	78.6	%
BMI	29.4 (8.7)	29.4 (9.2)	29.7 (10.1)	29.6 (8.5)	30.5 (10.9)	kg.m^-2^
OI on admission	92.7 (54.5)	92.7 (55.4)	97.0 (51.4)	104.4 (48.9)	92.0 (66.9)	mmHg
Smoking status	16.5	17.1	15.4	15.4	9.1	%
Arterial hypertension	55.9	52.2	63.6	75.0	78.6	%
Diabetes mellitus	23.4	21.2	27.3	39.3	28.6	%
COPD	5.1	4.4	6.1	14.3	0.0	%
Chronic heart failure	5.4	3.7	9.1	14.3	14.3	%
Coronary artery disease	8.1	7.7	3.0	14.3	14.3	%
Arrhythmia	6.2	5.1	15.2	10.7	0.0	%
Stroke	2.4	1.7	6.1	7.1	0.0	%
PVD	2.4	1.0	3.0	14.3	7.1	%
Liver disease	3.2	3.0	6.1	3.6	0.0	%
Malignancy	5.4	4.7	6.1	10.7	7.1	%
Connective tissue disease	4.0	4.4	6.1	0.0	0.0	%
Peptic ulcer disease	3.5	3.4	6.1	3.6	0.0	%
CKD Stage 1-2	2.9	0.3	9.1	14.3	21.4	%
CCI	2.0 (2.0)	2.0 (2.0)	3.0 (3.0)	3.0 (2.25)	2.5 (1.0)	

BMI: body mass index; COPD: chronic obstructive pulmonary disease; PVD: peripheral vascular disease; CKD: chronic kidney disease; CCI: Charlson comorbidity index.

### Kidney function during ICU stay

**Figure 1.**
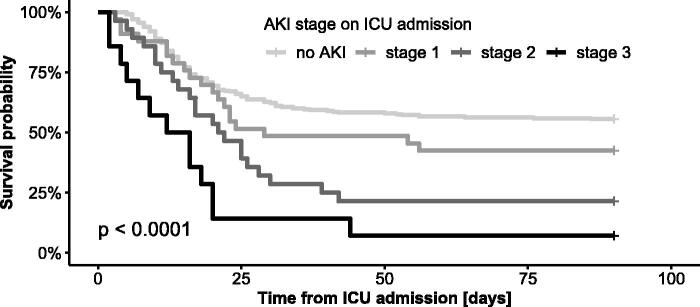
Kaplan–Meier survival curves for 90-day mortality from ICU admission stratified by AKI stage on ICU admission. No data were censored before Day 90.

**Figure 2.**
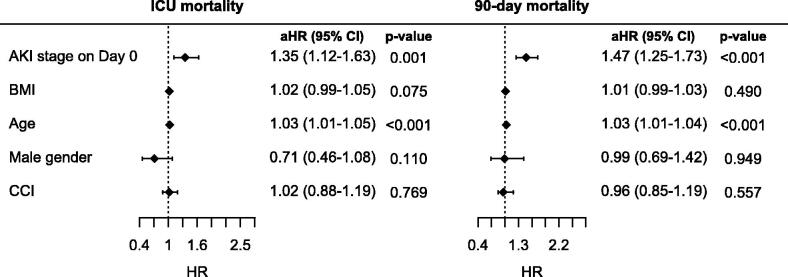
Cox regression analysis of the impact of AKI stage on ICU admission on ICU mortality and 90-day mortality adjusted for age, BMI, sex and comorbidities. HR, hazard ratio; aHR, adjusted hazard ratio; CCI, Charlson comorbidity index.

Mortality and the need for RRT increased with advanced AKI stages on ICU admission (Table 2, Figure 1). The length of stay did not differ according to the AKI stage on ICU admission. The AKI stage on admission remained a significant predictor of both ICU and 90-day mortality after adjustment for age, sex, BMI and comorbidities using the Cox regression model (Figure 2).

**Table 2.** Mortality and the need for RRT depending on AKI stage on ICU admission.

AKI stage	n	RRT	ICU mortality	90D mortality
All	372	17.5	38.4	50.0
no AKI	297	10.8	34.7	44.4
1	33	21.2	39.4	57.6
2	28	50.0	57.1	78.7
3	14	85.7	78.6	92.9
*p value*		*<0.001*	*<0.001*	*<0.001*

Values are expressed in percents; *p* values result from log-rank tests.

Table 3 describes the development of AKI stages between Day 0 and Day 2 of the ICU stay. Six percent of the patients without AKI on ICU admission developed AKI by Day 2. Thirty percent of the patients with AKI on ICU admission deteriorated into higher AKI stages.

**Table 3.** Development of AKI stage from Day 0 to Day 2. The values indicate numbers of patients with corresponding AKI stage on Day 0 (in rows) and on Day 2 (in columns).

AKI stage		Day 2				
		no AKI	1	2	3	Σ
Day 0	no AKI	279	10	4	4	297
	1	15	7	6	5	33
	2	4	9	8	7	28
	3	1	0	0	13	14
	Σ	299	26	18	29	372

The patients whose AKI stage increased from ICU admission to Day 2 had significantly worse outcomes than those with no change or who had a decrease in their AKI stage (Table 4). Eight patients in whom RRT was initiated within 48 h of ICU admission were excluded from this part of the analysis.

**Table 4.** Association of mortality and the need for RRT with the change in AKI stage from Day 0 to Day 2.

change in AKI stage	n	RRT	ICU mortality	90D mortality
increase	36	47	58	75
no change	299	11	35	46
decrease	29	21	34	52
*p value*		*<0.001*	*<0.001*	*<0.001*

Values are expressed in percents; *p* values result from log-rank tests.

**Figure 3.**
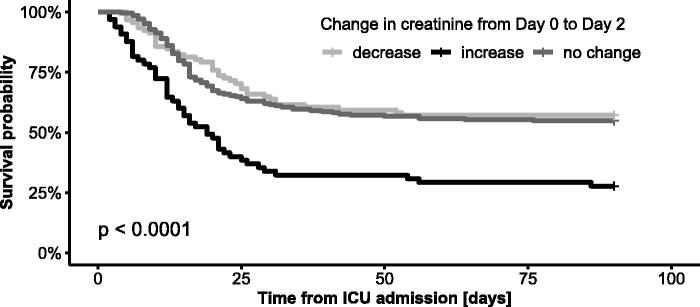
Kaplan–Meier survival curves for 90-day mortality from ICU admission stratified by change in creatinine within 48 h of ICU admission by more than 20%. No data were censored before Day 90.

**Figure 4.**
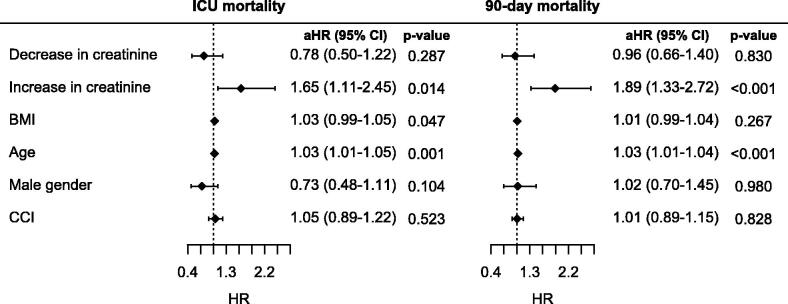
Cox regression analysis of the impact of change in creatinine within 48 h of ICU admission on ICU and 90-day mortality after being adjusted for age, BMI, gender and comorbidities. Stationary creatinine was used as a reference. The decrease and increase in creatinine were defined by a change in creatinine of more than 20%. HR: hazard ratio; aHR: adjusted hazard ratio; CCI: Charlson comorbidity index.

Similarly, the patients with creatinine increases of more than 20% from ICU admission to Day 2 had significantly worse outcomes than those who remained stationary (D2/D0 0.8-1.2) or improved (D2/D0 < 0.8) (Table 5, Figure 3). The change in creatinine within 48 h of ICU admission by more than 20% remained a significant predictor of both ICU and 90-day mortality after adjustment for age, sex, BMI and comorbidities using the Cox regression model (Figure 4). Eight patients in whom RRT was initiated within 48 h of ICU admission were excluded from this part of the analysis as well.

**Table 5.** Association of mortality and the need for RRT with a change in creatinine within 48 h of ICU admission by more than 20%.

change in creatinine level	n	RRT	ICU mortality	90D mortality
increase	61	38	57	70
no change	211	12	35	45
decrease	92	10	29	42
*p value*		*<0.001*	*<0.001*	*<0.001*

Values are expressed in percents; *p* values result from corresponding log-rank tests.

No significant relationship between the time to RRT initiation and ICU and 90-day mortality was found. The time to RRT initiation from ICU admission was 5.5 (10) days for the ICU survivors, 7 (8) days for the ICU nonsurvivors (*p* = 0.831) and 9 (9) and 6 (8) days for the 90-day survivors and nonsurvivors (*p* = 0.147).

The need for RRT was strongly associated with mortality (Table 6). Out of the 65 patients treated with RRT in the ICU, 18 patients (28%) survived up to ICU discharge, and 10 (15%) of them were alive at 90 days. In the patients requiring RRT, concomitant support with ECMO did not result in an additional increase in mortality. The patients treated with RRT alone had higher mortality than the patients treated with ECMO alone (90-day mortality 88% vs. 57%, *p* = 0.17).

**Table 6.** Association of mortality with the need for RRT and ECMO.

RRT	ECMO	n		ICU mortality		90D mortality	
yes	Yes	65	22	72	73	85	77
	No		43		72		88
no	Yes	307	64	31	50	43	57
	No		243		26		39
*p value*				*0.0002*		*0.0015*	

Values are expressed in percents; *p* values result from Fisher exact test.

### AKI analysis of COVID-19 waves

**Figure 5.**
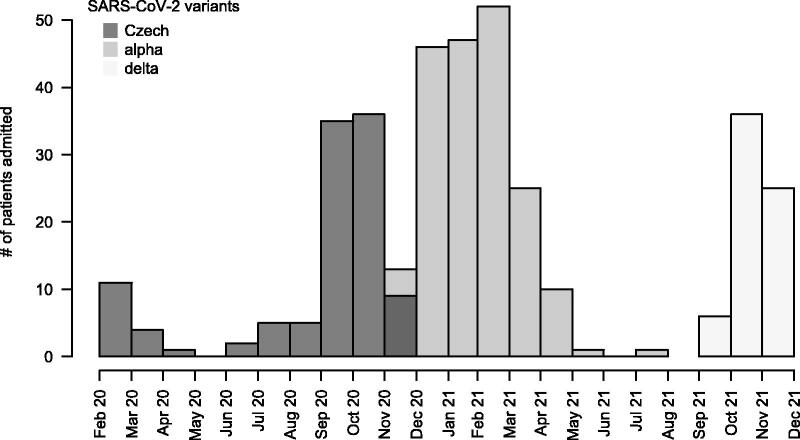
Number of COVID-19 ICU admissions per month.

The number of ICU admissions during the study period is shown in Figure 5. In agreement with worldwide and local epidemiologic situations, three consecutive COVID-19 waves were identified, which roughly corresponded with the prevailing SARS-CoV-2 virus variants.

No significant difference among the three COVID-19 waves with respect to the need for RRT and mortality was identified, although the patients in the third wave tended to have a lower mortality (Table 7). The patients requiring RRT had a significantly lower mortality during wave 3 than during waves 1 and 2. In contrast, mortality in the patients without RRT was comparable in all three waves. The prognostic significance of an increased D2/D0 creatinine ratio was present and comparable in all three periods.

**Table 7.** Development of need for RRT and mortality over three waves of COVID-19 in all patients and in patients requiring RRT.

COVID-19 wave	SARS-CoV-2 variant			ICU mortality		90D mortality	
		n	RRT	all	RRT	all	RRT
03-12/2020	Czech	108	18.5	38.9	75.0	53.7	90.0
01-08/2021	alpha	195	16.4	41.0	87.5	50.3	90.6
09-12/2021	delta	69	18.8	30.1	30.8	43.5	61.5
*p value*					*0.0002*		*0.180*

Values are expressed in percents; *p* values result from Fisher exact test.

### Comparison of AKI in ICU patients in the pre-COVID-19 and COVID-19 eras

ICU mortality (34.6% vs. 38.4%) as well as the need for RRT (15. % vs. 17.5%) was not significantly different between our pre-COVID-19 and COVID-19 groups of patients. However, the ICU mortality of the patients who required RRT was much higher in the COVID-19 group than in the pre-COVID-19 group (72.3% vs. 38.3%, *p* = 0.0004). On the other hand, the ICU mortality of the patients who did not require RRT was comparable in both eras (34.0% vs. 30.9%).

## Discussion

In our group of mechanically ventilated patients with severe COVID-19 pneumonia, a higher AKI stage on ICU admission resulted in more frequent RRT and higher mortality. Worsening of the AKI stage as well as an increase in creatinine during the first 2 ICU days signaled a highly increased mortality.

High mortality of critically ill COVID-19 patients with advanced AKI or the need for RRT was also found in other studies. RRT was used in 10-31% of patients, with an ICU mortality of patients requiring RRT of 50-70% [14,27–29], which is comparable with our results. Numerous studies have also shown the association between AKI on ICU admission and mortality in critically ill COVID-19 patients [14–19], although they differed substantially in the patient population included and in the outcome. For instance, 90-day mortality in patients without AKI on admission was reported to be 7–15% versus 44% in our study, but only 70-81% of that study’s patients were ventilated, compared with the 100% in our study. In contrast, Bezerra [15] reported an overall mortality of 90%; however, 79% (versus 23% in our study) of the patients received RRT, and 90% were ventilated.

Patients admitted with normal kidney function rarely deteriorate early in the ICU (6%), which is in contrast to patients admitted with AKI (30%). An early increase in AKI stage predicts a much higher need for RRT and mortality. Similar prognostic information is provided by the D2/D0 creatinine ratio. In our study, only 11% of the patients in whom D2/D0 remained stable or improved required RRT, compared to nearly 40% of patients whose D2/D0 increased. The ICU mortality of those with D2/D0 progression was 70% higher. The D2/D0 ratio appears to be a more sensitive parameter to detect subtle but clinically significant changes in kidney function than the change in AKI stage and is able to identify a larger group of patients (61 vs. 36 patients) at risk of RRT need or increased mortality. For example, the rather robust AKI stage classification cannot reflect a further increase in creatinine at stage 3, and generally, a higher change in creatinine is required to shift patients from a given AKI stage to another.

We observed no significant relationship between the day of RRT initiation and mortality. A similar result was published by Xu [30]. This is in contrast to the study of Qian [31], where early initiation of RRT in COVID-19 patients with severe AKI signaled better prognosis. Nevertheless, the data in this field are inhomogeneous for both COVID-19- and non-COVID-19-related AKI [32,33].

The need for RRT was the same in all three waves of COVID-19. RRT also remained a high-risk factor in all three waves and was associated with a twofold increase in mortality. In our cohort, the mortality of the patients requiring RRT even surpassed that of the ECMO patients.

Analysis of the three COVID-19 waves, roughly reflecting the various SARS-CoV-2 variants, revealed a signal of better outcome of the third wave patients with the prevailing delta variant. However, factors other than a change in the virus variant may have contributed to the improved outcome. Although our general therapeutic approaches, the selection and spectrum of patients admitted to the ICU or the indications for RRT have not changed much over time, new therapeutic options, such as antiviral drugs, have emerged. Better selection of patients suitable for ECMO support might also have contributed. Comparison with the patient population of 2022 with solely omicron variants would be interesting, but the low number of critically ill patients with omicron variants does not yet al.low us to perform a representative analysis. Corriero [34] compared delta and omicron variant profiles and found a much lower occurrence of AKI in the omicron variant (10% vs. 38%). However, he found a comparable mortality, mainly because of more frequent comorbidities in patients with the omicron variant.

The comparison of pre-COVID-19 and COVID-19 patients only stresses the significance of kidney compromise in COVID-19 pneumonia patients, possibly because of direct viral damage of the kidneys [9]. Detailed comparison of the significance of kidney dysfunction in COVID-19 patients with different primary pulmonary diseases would require a matched-paired analysis, which is beyond the aims of this study.

Our study has several limitations. First, we used a modified definition of AKI. The aforementioned studies [14–19] used the standard KDIGO definition [20], defining AKI as a proportional increase in creatinine compared to the baseline value or an absolute increase in creatinine or low diuresis within 48 h of admission. However, such a definition does not allow us to assess the impact of the early development of renal dysfunction within the first 48 h, which was the primary objective of our study, because we can only determine whether a patient has AKI on admission after the patient has been admitted for 48 h. In addition, we did not have available prehospital baseline creatinine values in most patients. Therefore, we chose a modified definition of AKI, where we calculated the expected normal creatinine by the MDRD equation and related the actual creatinine to this value. In this way, we can define the presence of AKI at the time of admission. For the same reason, we did not include diuresis in the definition, which, according to the KDIGO definition, requires measurement of diuresis up until 24 h. The disadvantage of our modified definition is its lower sensitivity for the detection of AKI. In our study, 20% of the patients had AKI on admission, compared with 32%, 36% and even 54% in the studies of Aukland [14], Lumlertgul [18] and Ghosn [16].

Second, estimation of baseline creatinine by the MDRD equation [20] introduces the possibility of misclassification of unidentified CKD as AKI, which overestimates the AKI stage [21]. However, it is an accepted method in cases of unknown baseline creatinine values [20,22]. We used a glomerular filtration rate of 100 mL/min in the equation, as this value provides the best baseline creatinine estimate to classify AKI in the setting of acute infection [22].

Finally, we chose a rather arbitrary threshold of 20% for a significant change in creatinine. The choice was based on the fact that this value exceeds the diurnal variation in creatinine, which is rarely greater than 10% in healthy subjects [35], as well as the measurement error, which is less than 5% in our laboratory.

## Conclusion

Our analysis of a retrospective single-center cohort of patients with severe COVID-19 pneumonia confirmed the prognostic significance of AKI on ICU admission as well as deterioration of kidney function during the first two ICU days.

## Ethics approval and consent to participate

The study protocol was approved by the ethics committee of the St. Anne’s University Hospital, Brno. This study was conducted in accordance with the ethical guidelines of the Declaration of Helsinki. The ethics committee waived the requirement for informed consent because the study only applied a retrospective analysis of anonymized data.

## Data Availability

Analyses of all data performed during this study are included in this manuscript. The datasets used and analyzed during the study are available from the corresponding author on reasonable request.
